# The Puppies’ Age at Adoption Time Influences the Behavioral Responses of Adult Dog

**DOI:** 10.3390/vetsci12020176

**Published:** 2025-02-14

**Authors:** Raffaella Cocco, Francesca Arfuso, Sara Sechi, Giuseppe Piccione, Claudia Giannetto, Federica Arrigo, Maria Rizzo

**Affiliations:** 1 Department of Veterinary Sciences, Teaching Veterinary Hospital, University of Sassari, Via Vienna 2, 07100 Sassari, Italy; rafco@uniss.it (R.C.); sarasechilavoro@tiscali.it (S.S.); 2 Department of Veterinary Sciences, University of Messina, Polo Universitario dell’Annunziata, 98168 Messina, Italy; gpiccione@unime.it (G.P.); claudia.giannetto1@unime.it (C.G.); federica.arrigo@studenti.unime.it (F.A.); rizzom@unime.it (M.R.)

**Keywords:** human–animal relationship, dog adoption, post-acquisition, early separation, behavioral differences

## Abstract

Early socialization and appropriate adoption time are key factors in shaping canine behavior and welfare. Stress and behavioral challenges are major concerns for owners, often leading to abandonment. Stress can be assessed through physiological and behavioral indicators, with behavior being a noninvasive and practical measure. Canine development includes critical sensitive periods, particularly the socialization phase (3–12 weeks), when puppies form primary social bonds and adapt to novel stimuli. Lack of positive experiences during this period can lead to adult behavioral problems like fear, aggression, or anxiety. This study analyzed the behaviors of 107 dogs adopted at different ages (≤1 month to ≥4 months) and from varied origin contexts (shelters, breeders, private). Using the validated C-BARQ tool, the research found that the age at adoption significantly influenced behavior, with puppies adopted ≤1 or ≤2 months showing higher behavioral signs of fear, anxiety, and attachment and attention-seeking tendencies compared to those adopted later. The findings highlight the importance of socialization and appropriate adoption timing to prevent behavioral issues. The study underscores the value of educating owners and shelters about expected behavioral changes and the benefits of professional behavioral assessments. By fostering realistic expectations and proactive interventions, these insights aim to improve canine welfare and reduce abandonment rates.

## Simple Summary

Early socialization and appropriate adoption time are crucial for canine behavior and welfare. Stress and behavioral challenges, such as fear, aggression, or separation-related behaviors, often lead to abandonment. Canine development includes sensitive periods, particularly the socialization phase (3–12 weeks), when puppies adapt to novel stimuli and form bonds. This study in Sardinia analyzed 107 dogs adopted at various ages (≤1 to ≥4 months) and found that puppies adopted ≤1 or ≤2 months exhibited higher fear, anxiety, and attachment and attention-seeking behaviors. The findings stress the importance of proper socialization, adoption timing, owner education, and behavioral assessments to improve canine welfare and reduce abandonment.

## 1. Introduction

The different relationships, the type of socialization, how this is established, and the time of adoption have an important influence on the behavior and well-being of dogs. This can lead to fear and aggression, and this then causes concern in owners. Other behaviors such as separation problems, domestic soiling, and destructive situations lead to abandonment or giving up dog ownership [1]. Stress levels in pets can be assessed using various parameters, with physiological and behavioral indicators being the most commonly employed. In this regard, behavioral parameters, which, can be measured easily and noninvasively, are of particular interest since they are especially useful for identifying stress and subsequent welfare concerns in dogs [2].

Behavior is the result of a series of neural and physiological processes that work together to produce responses to stimuli from the surrounding environment [3]. Indeed, precisely because dogs are highly sociable animals, successful communication (within the species, but especially with humans) is essential for them to sustain hierarchy and social bonds. In this respect, it is not surprising that dogs benefit similarly or more from the presence of a human being than from the presence of other dogs. Despite the domestic dog being widely regarded as a highly trainable species, thousands of dogs are abandoned in shelters each year due to perceived behavioral problems [4]. Alongside genetic and environmental factors, age plays a fundamental role in shaping canine behavior.

Canine development occurs in six sensitive periods, each critical to a dog’s behavioral development [5]. These stages include the prenatal period (the 9-week gestation), the neonatal period (birth to 2 weeks of age), the transition period (2–3 weeks), the socialization period (3–12 weeks), the juvenile period (12 weeks to 6 months), and the pubertal period (7–24 months) [6]. Among these, the socialization period is often described as a “critical period” for forming primary social relationships and attachments.

Primary social relationships are established differently depending on the period the puppies are experiencing. In the first three weeks, puppies show the ability to engage in intra- and inter-specific interactions and pro-social tendencies. In addition, feelings of fear and avoidance gradually decrease. From 3 to 12 weeks after birth, they develop an attachment to places and people [7]. During this time, puppies not only engage in pro-social behaviors with both conspecifics and humans but also exhibit reduced fear and avoidance in new situations. Between 3 and 5 weeks of age, puppies are more likely to approach unfamiliar people, although this tendency declines as they age. Failure to expose puppies to positive and stimulating experiences during this crucial window can result in adult behavioral problems, including separation anxiety and aggression [8].

Recent studies, based on behavior questionnaire answered by dogs’ owners, reveal that a significant portion of dogs display problematic behaviors, with many owners recognizing at least one behavioral issue in their pet [8,9,10]. Specifically, some researchers focused on the results obtained by a public, online questionnaire to capture up-to-date demographic and behavior problem metrics (i.e., fear/anxiety, aggression, jumping, excessive barking, coprophagia, obsessive-compulsive/compulsive behaviors, house soiling, rolling in repulsive materials, overactivity/hyperactivity, destructive behavior, running away/escaping, and mounting/humping) of 4114 dogs belonging to spanning mixed and pure breeds [9]. The study found a high prevalence (85%) of behavioral issues among dogs. Notably, factors such as sex, neuter status, origin, and lineage were found to influence the prevalence of these behavior problems [9].

Another study focused on the answers of dog owners on the prevalence, comorbidity, and breed specificity of seven canine anxiety-like traits (i.e., noise sensitivity, fearfulness, fear of surfaces and heights, inattention/impulsivity, compulsion, separation-related behavior, and aggression) showed that noise sensitivity and fear are the most common anxiety-related traits. When comparing the relative risk, the largest risk ratios were seen between hyperactivity/inattention, separation-related behavior, and com-pulsion and between fear and aggression. Furthermore, dog breeds showed large differences in prevalence of all anxiety-related traits, suggesting a strong genetic contribution [11]. Noteworthy, the perception about the dogs’ behavioral issues could be subjective and can change from one owner to another. More specifically, behaviors often labeled as “problematic” by owners, such as barking, digging, or chasing, may be perfectly normal for the dog within its environmental context. Though the behaviors of dogs may be perceived differently by their owners and the type of perception may influence the owner’s actual willingness to change those behaviors, it has been demonstrated that, among the behavioral categories, the aggressive canine behaviors were three times more likely to elicit an owner’s wish to address them [8]. It is of paramount importance to acknowledge that a dog’s behavior can significantly influence the dynamics of the dog–owner relationship, potentially leading to negative outcomes for both the animal and those around it [2]. In fact, it is estimated that up to 90% of dogs exhibit behaviors considered unacceptable by their owners, including noise sensitivity, fear, separation-related behaviors, inattention, aggression, hyperactivity/impulsivity, and compulsive behaviors [12]. Even when not pathological, these behaviors can adversely affect the dog’s well-being, strain the relationship with the owner, and, in extreme cases, result in abandonment, surrender to shelters, or euthanasia, posing a significant animal welfare issue.

To better understand and manage such issues, temperament tests are frequently used to evaluate and predict canine behavior. These tests are generally considered reliable tools for assessing how dogs respond to various stimuli encountered in daily life and for gauging their ability to cope with challenging or threatening situations [13,14,15]. One of the most widely utilized tools is the Canine Behavioral Assessment and Research Questionnaire (C-BARQ), developed by Hsu and Serpell in 2003 [16]. The C-BARQ has been translated and validated in several languages. This validated instrument has been instrumental in classifying behavioral phenotypes, tracking behavioral changes, and assessing the temperaments of guide and service dogs. The C-BARQ consists of 100 questions grouped into 14 factors, covering various behaviors, including types of aggression, fearfulness/anxiety, trainability, excitability, predatory chasing, and attachment/attention-seeking. Additionally, there are 27 miscellaneous items addressing behaviors like coprophagia and stereotypic spinning/tail-chasing.

In light of these considerations, the present study examined the potential factors influencing behavioral changes to better understand when and how these shifts occur. The survey focused on Sardinian dogs adopted at different ages and from various origin contexts (shelter, breeder, private), using the standardized and validated C-BARQ to track and monitor their behavior.

## 2. Materials and Methods

In this study, the authors gathered data on the prevalence of owner-reported potentially problematic behaviors in adult dogs. For this study, we included 107 dogs (ranging from 1 to 16 years old; 64 males, 42 females) of various breeds. All the animals were kept as pets. This study recruited owners of dogs presented for routine screening and prophylaxis at the Veterinary Teaching Hospital (VTH) of the University of Sassari (Sardinia, Italy). All dog owners provided informed consent prior to participating. Participation in the study was voluntary, and respondents were free to withdraw either by simple declaration or by not answering the survey by e-mail. The inclusion criteria for the study were as follows: (1) Participants must be residents of Sardinia, Italy; (2) be at least 18 years old; (3) belong to a family rather than being a single individual; (4) have only one pet in the household; (5) own a dog over one year old at the time of registration. Additionally, (6) participants had prior experience owning a dog. Furthermore, dogs were included in the study if they exhibited the following criteria: owner’s informed consent for the scientific use of their animal’s data; clinically healthy to physical exam, free from external and internal parasites, and in good nutritional condition; hematological and biochemical profile checked at the time of hospital admission. The dogs had been separated from their dam and littermates at various ages (≥4 months or ≤1 month, ≤2 months, and ≤3 months) and had been acquired through breeders, private sellers, or shelters. During the spring season of the year 2023, the study was performed in the northern part of Sardinia (Italy) (mean ambient temperature 11.7 ± 2.5 °C, mean relative humidity 78% ± 1.9%). In fact, climate change can significantly impact the health and well-being of pets. Reporting temperature and humidity is crucial because these factors directly affect an animal’s comfort, behavior, and risk of developing heat-related illnesses. High temperatures combined with elevated humidity can increase the likelihood of heat stress or heatstroke, especially in certain breeds or older pets. The clinical and laboratory examinations, including routine hematology (i.e., red blood cell, hemoglobin concentration, hematocrit, mean corpuscular volume, mean corpuscular hemoglobin, mean corpuscular hemoglobin concentrations, platelet and white blood cell) and biochemistry (i.e., total proteins, albumin, globulins, glucose, triglycerides, amylase, lipase, cholesterol, alkaline phosphatase, creatine phosphokinase, urea, creatinine, total bilirubin, gamma-glutamyl transferase, aspartate aminotransferase, alanine aminotransferase, calcium, phosphorus) profiles performed revealed that the dogs were free of external and internal parasites, in good nutritional condition and pathologies were excluded as a possible primary or secondary cause of the behavioral problems. To assess health status, behavior was observed, heart rate as well respiratory profile, cough, nasal discharge, eye discharge, appetite, and fecal consistency were measured, and the umbilicus examined. All dogs were fed twice a day with high-quality commercial diets in accordance with their body size and age. Each procedure was performed in accordance with the standards recommended by Directive 2010/63/EU on the protection of animals used for scientific purposes. The recommendations of the ARRIVE guidelines were also consulted. The 100-question version of the C-BARQ was used to evaluate behavior changes [15,16]. Owners scored each behavior on a five-point Likert scale from 0 to 4. C-BARQ subscales for all four timepoints were compiled based on previously published behavior traits: training and obedience, aggression, fear and anxiety, separation-related behavior, excitability, attachment and attention-seeking, and miscellaneous. For parameters such as sex, age, type of breeding origin context, and age of adoption, a type of analysis known as descriptive statistical analysis was performed in order to test the characteristics of the data distribution. For all data obtained, a Shapiro–Wilk test was performed to verify the normality of the distribution. Data were not normally distributed (*p* < 0.05). A Kruskal–Wallis’s test, followed by Dunn’s post hoc comparison test, was applied to determine the statistical effect of breeding origin type and age of adoption on studied behavior traits. *p* values < 0.05 were considered statistically significant. Statistical analysis was performed using the statistical software Jamovi (Computer Software Version 2.6).

## 3. Results

For this study, 107 dogs (range 1–16 years; 65 (60.7%) intact males, 42 (39.3%) intact females) were included. The study population of dogs was divided into groups according to the breeding origin type before adoption (breeders, private sellers, or shelters) and age of adoption (≤1 month, ≤2 months, ≤3 months, or ≥4 months). Table 1 showed signalment data (breed, age expressed in years, and gender) for enrolled dogs divided into groups according to the breeding origin type (breeders, private sellers, or shelters).

**Figure 1:**
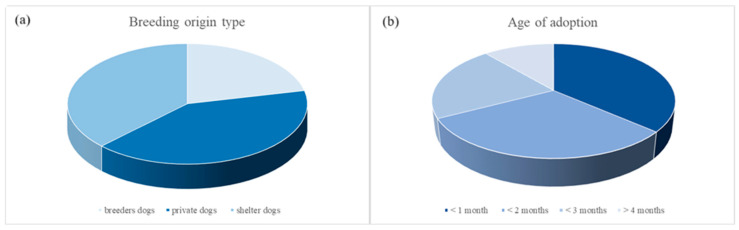
Graphical distribution of dogs population according to the breeding origin type (**a**) (breeders, private sellers, or shelters) and age of adoption (**b**) (≤1 month, ≤2 months, ≤3 months, or ≥4 months).

Regarding the breeding origin type, descriptive statistical analysis showed: 23 breeders dogs (21.5%), 44 private dogs (41.1%), 40 shelter dogs (37.4%) (Figure 1a). Regarding the age of adoption, descriptive statistical analysis showed: 39 dogs adopted at <1 month (36.4%), 33 dogs adopted at <2 months (30.8%), 23 dogs adopted at <3 months (21.5%), and 12 dogs adopted at >4 months (11.2%) (Figure 1b).

**Figure 2:**
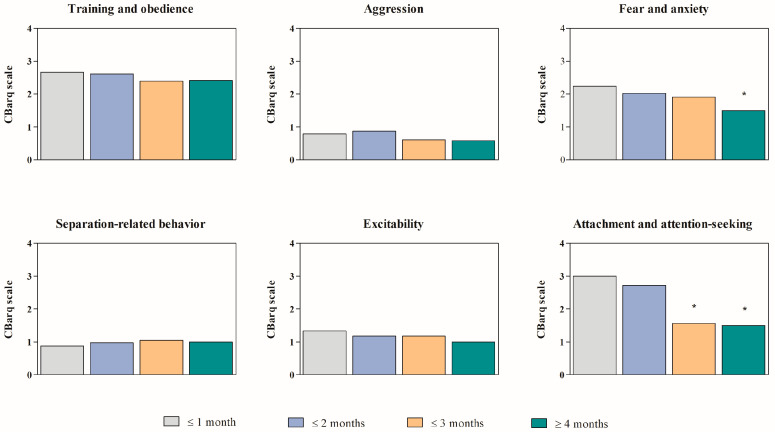
The prevalence of behavior traits among different age of adoption across various categories in dogs included in the study. Significances: (effect of time of adoption) * vs. <1 month and <2 months.

After reviewing several questionnaires, we noticed that owners often skipped section Miscellaneous, likely because they found it too repetitive. As a result, this section was excluded from the statistical analysis. The Kruskal–Wallis’s test showed that the management contexts of puppies before adoption did not affect the behavior of dogs, whereas the dogs aged ≤1 and ≤2 months at the time of adoption exhibited different patterns of behavioral reaction than puppies aged ≤3 and ≥4 months at the time of adoption, in section Attachment and attention-seeking (Figure 2). In the section Fear and anxiety, the statistical analysis also showed that the dogs aged ≤1 and ≤2 months at the time of adoption exhibited higher patterns of behavioral reaction than puppies aged ≥4 months at the time of adoption (Figure 2). No statistically significant differences were observed in other sections of the questionnaires. Tables S1 and S2 (Supplementary Materials) present the frequency and percentage distribution of behaviors across various categories in dogs included in the study. These behaviors are segmented by behavioral scales and are analyzed according to the dogs’ adoption age and the origin contexts of the puppies before adoption.

## 4. Discussion

The well-being of dogs is multidimensional, encompassing physical, relational, and motivational aspects. Based on these dimensions, every owner should guide their actions and decisions to ensure the well-being of the dog [17,18]. Most problematic behaviors arise from the normal regulatory processes of species-typical behavior. The issue is not the animal’s behavior itself but rather the challenges it poses for its owner. Preventing undesirable behaviors is essential, not only because many owners who surrender their dogs might choose to keep them if these issues were resolved, but also because behavioral problems are often associated with increased anxiety levels, which can significantly impact the dog’s welfare [9]. It is known that the cross-talk among genetics, environment, and experience contributes to the development of most aspects of the behavior of the animal. As a matter of fact, the combination of these factors, operating in a transactional fashion over time, could have neuroendocrine, behavioral, or epigenetic consequences on the physiology and behavior of puppies, which could persist throughout life [17]. Behavioral conditions such as anxiety/fear, noise phobia, conspecific aggression, impulse/control aggression, predatory aggression, and obsessive–compulsive disorder in dogs may appear at between one and two years of age; however, the social maturity period when neural systems are undergoing extensive developmental changes in puppies may become evident as early as three months of age [18,19]. A puppy’s ability to face new situations, whether derived from physical or social environmental stimuli, lays the foundation for healthy behavior with both conspecifics and humans [18,19,20]. This ability is fundamental to the overall well-being of the animal. The results of this study highlight the critical importance of adoption age and the socialization period in the behavioral development of dogs. Specifically, puppies adopted at an early age (≤1 or ≤2 months) exhibit specific behavioral tendencies, such as higher levels of fear and anxiety, combined with increased behavioral signs of attention-seeking towards their owners that may reflect insecure attachment. As a transient emotional response triggered by specific stimuli, fear is a fundamental survival mechanism conserved across species, enabling individuals to react appropriately to threatening situations. However, fearfulness can be a distinct personality trait, and when fear becomes excessive, prolonged, or generalized, it develops into a behavioral disorder that can severely impact a dog’s well-being. Chronic fear and anxiety can interfere with normal functioning, lead to heightened distress, increase susceptibility to various health issues, and even reduce lifespan [21]. While the study primarily suggests that adoption at an early age may predispose dogs to certain behavioral problems, the data on the level of socialization before or after adoption are not available; thus, this could represent a limitation of the study. Future research should aim to address this gap by assessing the quality and frequency of social interactions during early development. The preliminary findings gathered in the current study suggest that premature separation from the mother and littermates can interfere with the normal development of social skills, emotionality, and general adaptability in puppies, potentially leading them to demonstrate extreme neophobic responses. Considering that behavior problems could lead to distress for owners, veterinarians should be prepared to offer advice and provide information to owners to prevent damage to the human-pet relationship, which may cause dogs to be relinquished to shelters [22]. This is essential to prevent breakdowns in the human–animal bond, which may ultimately result in dogs being relinquished to shelters [22]. The consciousness that early separation from the litter exerts influence on specific and problematic behavioral patterns in dogs might give information that will improve behavioral intervention for owners of early separated puppies. Anyway, it is strongly suggested that the adoption occur when the puppy has had time to benefit from socialization with its mother and littermates, typically around 2–3 months of age [22]. Puppies separated from the litter before 60 days of age tend to display problematic behaviors, such as fear during walks, excessive barking, and destructive behavior [23,24,25]. The scientific literature supports these conclusions, indicating that the socialization period is crucial for developing behavioral resilience. The time window between 3 and 16 weeks of life is particularly significant for acquiring social skills and adapting to new stimuli [26,27,28]. Puppies that do not receive adequate stimuli during this phase may develop behavioral difficulties in adulthood, such as fear, anxiety, and, in extreme cases, aggressive reactions [21]. Therefore, introductions to new stimuli should be tailored to the puppies’ developmental stage and presented at an intensity that does not trigger avoidance, preventing them from becoming overwhelmed [20,27]. While dogs exhibit behavioral changes throughout their lives, puppies are especially sensitive to environmental influences during the socialization period, making them more likely to develop specific responses and preferences compared to other stages of development [20]. However, the role of the physical and social environment, diet, and individual characteristics was not investigated in this study. This lack of data limits the ability to fully understand the implications, highlighting the need for future research that incorporates these factors to strengthen and expand the findings. Another relevant aspect is the role of the post-adoption environment and the skills of the owners. Although the pre-adoption origin context did not have a significant impact on dog behaviors in this study, the environment provided by new owners can significantly influence adaptation success. For example, dogs predisposed to fear or anxiety may benefit from a structured and predictable environment, along with targeted interventions to promote progressive socialization. Exploring correlations between the frequency of social activities, dietary patterns, and emotional states could further enhance the understanding of these phenomena. In this study, the pre-adoption origin context (breeders, private sellers, or shelters) did not significantly influence dog behaviors. However, the study results also suggest that providing information and training to owners could be key tools in improving the well-being of adopted dogs. Offering future owners a clear understanding of realistic expectations and potential behavioral challenges associated with early-age adoption can help reduce the risk of returns, which often represent a humane solution to unsuitable adoption rather than an act of abandonment [19]. In this context, validated tools such as C-BARQ can play a fundamental role in monitoring behaviors, enabling timely intervention to mitigate emerging problems [9,12]. Although it is widely accepted among behavioral scientists that C-BARQ is a valuable tool for studying animal behavior, we believe that one criticism of the test is its complexity, which may discourage many people from using it. In our view, it could be simplified into two main categories: (1) attention and curiosity and (2) fear, anxiety, and dependence, as the other measured characteristics are largely related to these criteria. An examination of this might be useful for putting the suggestions into practice which would be useful for the welfare of dogs and their owners. From a practical perspective, shelters and breeders should carefully consider adoption age as a priority criterion to ensure the successful integration of the dog into its new family. Delaying adoption until the puppy has completed a significant portion of the socialization period may increase the likelihood of positive adaptation [12,22,28]. Additionally, implementing educational programs for new owners to promote proper and informed management of adopted dogs could reduce the frequency of behavioral problems. Another practical implication involves investing in assessment and behavioral intervention programs in shelters to identify potential challenges early and provide personalized support. These interventions could include controlled socialization, gradual exposure to new stimuli, and specific behavioral training to facilitate dog adoption. Finally, the results raise important ethical considerations regarding the origin and adoption of puppies. Ensuring that every dog has the opportunity to develop its social skills and live in an environment that meets its motivational needs is a shared responsibility among breeders, shelters, and owners [23,27,28]. Adopting a holistic, evidence-based approach to canine behavior management could significantly improve animal welfare and reduce the burden on shelters.

## 5. Conclusions

In conclusion, this study provides valuable insights for veterinarians, behavioral professionals, and shelters, suggesting that conscious adoption, supported by educational interventions and proper management of the socialization period, is crucial to ensuring the long-term well-being of dogs. Implementing strategies based on this evidence could lead to significant improvements in human–animal relationships and a reduction in behavioral problems, benefiting both dogs and their adoptive families. However, while the age of adoption appears to be a key factor, many other potentially relevant factors, not herein assessed, are worthy of investigation in further studies.

## Figures and Tables

**Table 1:** Signalment data for enrolled dogs divided into groups according to the breeding origin type (breeders, private sellers, or shelters).

Breed	N (%)	Age	Gender	N
Breeders dog	23 (21.5%)			
Rottweiler,	5	Median 5 years	Males	14
Mixed	4	Range 1–16	Females	9
German shepherd	3	Age of adoption		
Chihuahua, Cocker spaniel, Jack Russel terrier (2 each)	6	≤1 month		4
		≤2 months		10
Poodle, Maltese, Labrador retriever, Dachs hound, Dobermann (1 each)	5	≤3 months		5
		≥4 months		4
Private dog	44 (41.1%)			
Mixed	11	Median 4 years	Males	27
Labrador retriever,	5	Range 1–10	Females	17
German shepherd	4	Age of adoption		
Dachs hound, Boxer, Cocker spaniel, Jack Russel terrier (3 each)	9	≤1 month		17
		≤2 months		17
American Staffordshire terrier, Pitbull, Chihuahua, Poodle (2 each)	8	≤3 months		7
		≥4 months		3
Beagle, Dobermann, Dalmatian, Pointer, Italian rough-haired segugio, Yorkshire terrier, English setter (1 each)	7			
Shelter dogs	40 (37.4%)			
Mixed	34	Median 4 years	Males	24
English setter	2	Range 1–12	Females	16
Dachs hound, Italian rough-haired segugio, Yorkshire terrier, Jack Russell terrier (1 each)	4	Age of adoption		
		≤1 month		7
		≤2 months		12
		≤3 months		12
		≥4 months		9

## Data Availability

The data presented in this study are available within the manuscript and supplementary materials.

## Supplementary Materials

The following supporting information can be downloaded at: https://www.mdpi.com/article/10.3390/vetsci12020176/s1, Table S1. Frequency and percentage distribution of behaviors across various categories in dogs included in the study, segmented by adoption age and behavioral scales. Table S2. Frequency and percentage distribution of behaviors across various categories in dogs included in the study, segmented by breeding origin type and behavioral scales.
