# Supplementary material for: The Puppies’ Age at Adoption Time Influences the Behavioral Responses of Adult Dog
Source: Vet Sci. 2025 Feb 14;12(2):176. doi: 10.3390/vetsci12020176 (PMC11860672; doi:10.3390/vetsci12020176)
Supplement: Supplementary file 1 [file vetsci-12-00176-s001.zip › vetsci-3451307-supplementary.pdf]

Table S1. Frequency and percentage distribution of behaviors across various categories in dogs included in the study, segmented by adoption age and behavioral scales.

		"Training and obedience"		"Aggression"		Fear and anxiety		Separation-related behavior		Excitability		Attachment and attention-seeking	
**Scale**	**Adoption age**	**Observed**	**% of total**	**Observed**	**% of total**	**Observed**	**% of total**	**Observed**	**% of total**	**Observed**	**% of total**	**Observed**	**% of total**
**0**	***< 1 month***	0	0.0%	12	11.2%	0	0.0%	12	11.2%	1	0.9%	0	0.0%
	< 2 months	0	0.0%	11	10.3%	0	0.0%	12	11.2%	0	0.0%	1	0.9%
	< 3 months	0	0.0%	12	11.2%	0	0.0%	6	5.6%	3	2.8%	8	7.5%
	> 4 months	0	0.0%	5	4.7%	0	0.0%	4	3.7%	2	1.9%	1	0.9%
**1**	***< 1 month***	0	0.0%	17	15.9%	0	0.0%	16	15.0%	23	21.5%	0	0.0%
	< 2 months	0	0.0%	22	20.6%	7	6.5%	19	17.8%	34	31.8%	3	2.8%
	< 3 months	0	0.0%	8	7.5%	4	3.7%	12	11.2%	15	14.0%	3	2.8%
	> 4 months	0	0.0%	7	6.5%	6	5.6%	5	4.7%	8	7.5%	5	4.7%
**2**	***< 1 month***	12	11.2%	3	2.8%	25	23.4%	2	1.9%	6	5.6%	10	9.3%
	< 2 months	15	14.0%	6	5.6%	24	22.4%	6	5.6%	3	2.8%	14	13.1%
	< 3 months	14	13.1%	3	2.8%	17	15.9%	3	2.8%	3	2.8%	5	4.7%
	> 4 months	7	6.5%	0	0.0%	6	5.6%	2	1.9%	2	1.9%	5	4.7%
**3**	***< 1 month***	20	18.7%	1	0.9%	8	7.5%	3	2.8%	3	2.8%	13	12.1%
	< 2 months	24	22.4%	0	0.0%	8	7.5%	1	0.9%	2	1.9%	9	8.4%
	< 3 months	9	8.4%	0	0.0%	2	1.9%	2	1.9%	2	1.9%	5	4.7%
	> 4 months	5	4.7%	0	0.0%	0	0.0%	1	0.9%	0	0.0%	1	0.9%
**4**	***< 1 month***	1	0.9%	0	0.0%	0	0.0%	0	0.0%	0	0.0%	10	9.3%
	< 2 months	0	0.0%	0	0.0%	0	0.0%	1	0.9%	0	0.0%	12	11.2%
	< 3 months	0	0.0%	0	0.0%	0	0.0%	0	0.0%	0	0.0%	2	1.9%
	> 4 months	0	0.0%	0	0.0%	0	0.0%	0	0.0%	0	0.0%	0	0.0%

Table S2. Frequency and percentage distribution of behaviors across various categories in dogs included in the study, segmented by breeding management type and behavioral scales.

		"Training and obedience"		"Aggression"		Fear and anxiety		Separation-related behavior		Excitability		Attachment and attention-seeking	
**Scale**	**Breeding management type**	**Observed**	**% of total**	**Observed**	**% of total**	**Observed**	**% of total**	**Observed**	**% of total**	**Observed**	**% of total**	**Observed**	**% of total**
**0**	***Breeders***	0	0.0%	10	9.3%	0	0.0%	10	9.3%	2	1.9%	5	4.7%
	Private	0	0.0%	11	10.3%	0	0.0%	8	7.5%	3	2.8%	3	2.8%
	Shelter	0	0.0%	19	17.8%	0	0.0%	16	15.0%	1	0.9%	2	1.9%
**1**	***Breeders***	0	0.0%	9	8.4%	7	6.5%	10	9.3%	14	13.1%	3	2.8%
	Private	0	0.0%	24	22.4%	4	3.7%	21	19.6%	28	26.2%	5	4.7%
	Shelter	0	0.0%	21	19.6%	6	5.6%	21	19.6%	38	35.5%	3	2.8%
**2**	***Breeders***	9	8.4%	3	2.8%	13	12.1%	2	1.9%	5	4.7%	4	3.7%
	Private	20	18.7%	5	4.7%	30	28.0%	5	4.7%	4	3.7%	14	13.1%
	Shelter	19	17.8%	4	3.7%	29	27.1%	6	5.6%	5	4.7%	16	15.0%
**3**	***Breeders***	14	13.1%	1	0.9%	3	2.8%	1	0.9%	2	1.9%	6	5.6%
	Private	19	17.8%	0	0.0%	6	5.6%	5	4.7%	5	4.7%	13	12.1%
	Shelter	25	23.4%	0	0.0%	9	8.4%	1	0.9%	0	0.0%	9	8.4%
**4**	***Breeders***	0	0.0%	0	0.0%	0	0.0%	0	0.0%	0	0.0%	5	4.7%
	Private	1	0.9%	0	0.0%	0	0.0%	1	0.9%	0	0.0%	5	4.7%
	Shelter	0	0.0%	0	0.0%	0	0.0%	0	0.0%	0	0.0%	14	13.1%
